# Vascular cell adhesion molecule 1: a marker for atrial fibrillation and heart failure risk

**DOI:** 10.1093/europace/euaf246

**Published:** 2025-10-25

**Authors:** Jonas Alexander Baadsgaard, Oliver Bundgaard Vad, August Krebs Hessellund, Søren Zöga Diederichsen, Christian Paludan-Müller, Jesper Hastrup Svendsen

**Affiliations:** Department of Cardiology, The Heart Centre, Copenhagen University Hospital—Rigshospitalet, Copenhagen, Denmark; Department of Biomedical Sciences, University of Copenhagen, Copenhagen, Denmark; Department of Cardiology, The Heart Centre, Copenhagen University Hospital—Rigshospitalet, Copenhagen, Denmark; Department of Biomedical Sciences, University of Copenhagen, Copenhagen, Denmark; Department of Cardiology, The Heart Centre, Copenhagen University Hospital—Rigshospitalet, Copenhagen, Denmark; Department of Cardiology, The Heart Centre, Copenhagen University Hospital—Rigshospitalet, Copenhagen, Denmark; Department of Cardiology, The Heart Centre, Copenhagen University Hospital—Rigshospitalet, Copenhagen, Denmark; Department of Cardiology, The Heart Centre, Copenhagen University Hospital—Rigshospitalet, Copenhagen, Denmark; Department of Clinical Medicine, Faculty of Health and Medical Sciences, University of Copenhagen, Copenhagen, Denmark

**Keywords:** sVCAM-1, Proteomics, Biomarker, Atrial fibrillation, Heart failure

## Abstract

**Aims:**

The plasma protein soluble vascular cell adhesion molecule 1 (sVCAM-1) has been suggested as a biomarker for atrial fibrillation (AF). This study aimed to evaluate sVCAM-1 as a marker of AF and heart failure (HF) risk in the UK Biobank, incorporating genetic risk.

**Methods and results:**

Participants were included from 2006 to 2010. End of follow-up was 2023. Outcomes were incident AF and HF. Hazard ratios (HRs) per standard deviation increase in sVCAM-1 were assessed using Cox proportional hazard regression models. In sub-analyses, the cohort was stratified by tertiles of polygenic risk score (PRS) of AF and sVCAM-1. Associations between sVCAM-1 and cardiac magnetic resonance imaging measures were assessed in a sub-cohort. Among 48 495 included individuals, 54.6% were women. Median age at enrollment was 58 (50–63) years. During follow-up, 3484 were diagnosed with AF and 1937 with clinically diagnosed HF. Increasing sVCAM-1 levels were associated with rates of AF [HR: 1.72, 95% confidence interval (CI): 1.54–1.91] and HF (HR: 2.04, 95% CI: 1.78–2.34). In the highest sVCAM-1 tertile, 10-year cumulative incidence for AF and HF were 6.44% (95% CI: 6.05–6.82) and 3.01% (95% CI: 2.74–3.29), respectively. Stratified by tertiles of AF PRS and sVCAM-1 levels, a dose–response-like relationship emerged. In the imaging sub-cohort (*n* = 933), higher sVCAM-1 levels were associated with a reduced LA_EF_ (β: −2.51, 95% CI: −4.33 to −0.70).

**Conclusion:**

Higher sVCAM-1 levels were associated with AF and HF and lower LA_EF_. Integration of an AF PRS with sVCAM-1 levels identified a dose–response-like relationship with risk of AF.

## Graphical Abstract

**Graphical Abstract:**
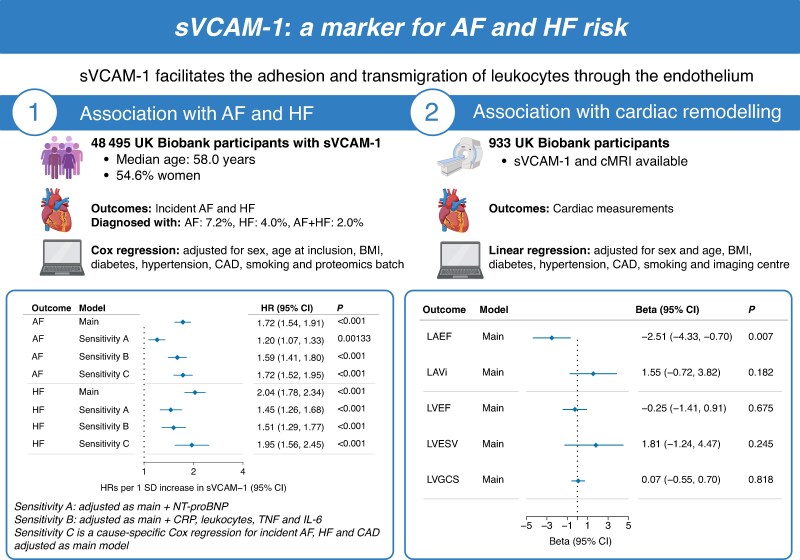
AF, atrial fibrillation; BMI, body mass index; CAD, coronary artery disease; CI, confidence interval; cMRI, cardiac magnetic resonance imaging; HF, heart failure; HR, hazard ratio; IL-6, interleukin-6; LA_EF_, left atrial emptying fraction; LAVi, left atrial volume index; LV_EF_, left ventricular ejection fraction; LV_ESV_, left ventricular end-systolic volume; LV_GCS_, left ventricular global circumferential strain; sVCAM-1, soluble vascular cell adhesion molecule 1; TNF, tumour necrosis factor. *Created in BioRender. Baadsgaard, J. (2025)*  https://BioRender.com/eomk29v.

What’s new?

- The plasma protein soluble vascular cell adhesion molecule 1 (sVCAM-1) is linked with inflammation and has been suggested as a potential biomarker for cardiovascular disease, including atrial fibrillation (AF) and heart failure (HF).
- Leveraging large-scale clinical data from the UK Biobank, we found that higher levels of sVCAM-1 were associated with increased rates of AF and HF, independently of known biomarkers of HF and inflammation. In the highest sVCAM-1 tertile, 10-year cumulative incidence for AF and HF were 6.44% (95% CI: 6.05–6.82) and 3.01% (95% CI: 2.74–3.29), respectively.
- Stratified by AF polygenic risk score (PRS) and sVCAM-1 levels, we observed a dose–response-like relationship with AF risk, with the highest risk in individuals in the top tertiles of both PRS and sVCAM-1.
- In a sub-cohort with both cardiac magnetic resonance imaging and sVCAM-1 measures, we found that higher sVCAM-1 levels were associated with reduced left atrial emptying fraction.

## Introduction

Atrial fibrillation (AF) is the most common arrhythmia, affecting almost 60 million people worldwide. In the coming decades, the prevalence of AF is expected to rise more than two-fold.^1–3^ It is associated with an increased risk of stroke, heart failure (HF), and premature death.^1,4^ Heart failure is the most common complication of AF, affecting two in five patients with AF.^5^ Heart failure affects more than 56 million people worldwide and is associated with a 5-year survival rate of only 50%.^6^ Heart failure also represents a substantial healthcare concern. Consequently, risk stratification and screening for AF are major research focuses. The results on the efficacy of screening programmes for AF have been conflicting.^7–9^ N-terminal pro–B-type natriuretic peptide (NT-proBNP) is regarded as the most well-established plasma protein biomarker for predicting AF and HF.^10–14^ The discovery of new biomarkers could improve risk stratification and screening,^15,16^ while also improving our understanding of the underlying pathology driving AF and HF.^17^

Vascular cell adhesion molecule 1 (VCAM-1) is a protein that plays an important role in acute and chronic inflammation, where it facilitates leukocyte adhesion and transmigration from the circulation through the endothelium, thereby promoting inflammation in the surrounding tissue.^18–20^ The soluble form sVCAM-1 can be measured in plasma. It has been established as a key contributor in the pathogenesis of atherosclerosis, and it is viewed as a potential anti-atherosclerotic drug target.^21^ Previous studies in moderately sized cohorts have also reported an association between increased levels of sVCAM-1 and AF.^22–24^

Leveraging and integrating clinical data and plasma proteomics from 48 495 individuals from the UK Biobank cohort, this study sought to assess associations between the plasma concentration of sVCAM-1 and incident AF and HF. By including a polygenic risk score (PRS) for AF, we investigated sVCAM-1 as a biomarker in combination with genetic predisposition for AF. As left atrial and left ventricular remodelling are increasingly recognised as parts of AF pathophysiology, we integrated cardiac magnetic resonance imaging (cMRI) in a sub-cohort to assess associations between sVCAM-1 and cardiac remodelling.

## Methods

### Study cohort

This study was conducted in the UK Biobank, a large biobank containing clinical and genetic data on more than 500 000 individuals from the United Kingdom. Individuals were enrolled between 2006 and 2010, and all participants provided written informed consent. The UK Biobank obtained approval from the North West Centre for Research Ethics Committee (#11/NW/0382). Details of the UK Biobank regarding methodology have been described previously by Bycroft *et al.*^25^

This study was conducted on a subset of individuals with available plasma proteomic measurements of sVCAM-1, and no prior history of HF or AF (*n* = 48 495). We excluded individuals with disagreement between reported sex and genetic sex, with missing data on body mass index (BMI) or with missing data on smoking status. Protein measurements were captured on the Olink Explore 3072 platform, and protein expression levels were transformed to a mean of zero and a standard deviation (SD) of one. In-depth methodology has previously been described by Sun *et al.*^26^

### Polygenic risk score

To calculate a PRS for AF, we obtained PRS weights published and validated by Weng *et al.*^27^ The PRS weights were calculated using the PRS-continuous shrinkage algorithm,^26^ based on summary statistics from a large genome-wide association study on AF.^28^ This study did not overlap with the UK Biobank cohort to avoid bias due to overfitting. We applied the PRS weights to all individuals and calculated the corresponding PRS using PLINK.^29^ The PRS was normalized by scaling to a mean of zero and an SD of one. To assess PRS and sVCAM-1 levels in aggregate, we stratified the cohort into nine groups based on tertiles of PRS and sVCAM-1 (low, intermediate, and high).

### Cardiac measures

A subset of the UK Biobank participants was invited to an additional follow-up visit to undergo a cMRI scan. In individuals where data on cMRI and new measures of sVCAM1 were available (*n* = 933), we assessed associations with left atrial emptying fraction (LA_EF_), left atrial volume index (LAVi) = left atrial maximum volume/body surface area, left ventricular ejection fraction (LV_EF_), left ventricular end systolic volume (LV_ESV_), and left ventricular global circumferential strain (LV_GCS_). LA_EF_, LV_EF_, and LV_GCS_ were measured in percentage (%). LV_ESV_ were measured in millilitre (mL). Left atrial volume index was measured in mL per square metre (mL/m^2^). The methodology for cardiac measurements has previously been described by Bai *et al.*^30^

### Outcomes and statistical analysis

We estimated hazard ratios (HRs) for incident AF and HF using multivariable Cox regression models. Index date was defined as the date of enrolment in the UK Biobank. Participants were followed until outcome, death, or end of follow-up (1 January, 2023), whichever came first. Models were adjusted for sex, age at inclusion, BMI, diabetes, hypertension, coronary artery (CAD) disease, smoking status, and proteomic batch (to reduce the impact of experimental variations). In sensitivity analysis A, we further adjusted for NT-proBNP, and in sensitivity analysis B, we further adjusted for the following inflammation markers: C-reactive protein (CRP), leukocyte counts, tumour necrosis factor (TNF), and interleukin-6 (IL-6). Definitions of outcomes, covariates, and corresponding UK Biobank data fields are summarized in Supplementary material online, *Table S1*. To assess potential nonlinear relationships, we performed penalized spline models with three degrees of freedom between sVCAM-1, as a continuous variable, and the outcomes. To assess potential associations, Pearson correlation coefficients were calculated between sVCAM-1 and the following biomarkers used as covariates in sensitivity analyses: NT-proBNP, CRP, leukocytes, TNF, and IL-6.

In a sub-cohort with exclusion of individuals with prior history of CAD, we performed sensitivity analysis C using a multivariable cause-specific Cox regression for incident AF, HF, and CAD, to ensure that our results were not merely driven by CAD. The models were adjusted for sex, age at inclusion, BMI, diabetes, hypertension, smoking status, and proteomic batch.

In a sub-analysis, we assessed concomitant AF and HF using Cox proportional hazards models with a time-dependent covariate. For the AF outcome, incident HF was considered as a time-dependent covariate and vice versa. Models were adjusted for sex, age at inclusion, BMI, diabetes, hypertension, CAD, smoking status, and proteomic batch.

In a sub-analysis on the entire cohort, we estimated HR for AF stratified by tertiles of PRS and sVCAM-1 levels using a multivariable Cox regression model adjusted for sex, age at inclusion, BMI, diabetes, hypertension, coronary artery disease, smoking status, and proteomic batch.

We calculated continuous net reclassification improvement (NRI) indices at 5 years to assess the added value of sVCAM-1 over NT-proBNP. Net reclassification improvement indices were constructed using the *nricens* package in R. We calculated event NRI (NRI^+^) as the net proportion of cases reclassified upwards versus downwards, and non-event (NRI^–^) as the net proportion of non-cases reclassified downwards versus upwards. The overall NRI was defined as the sum of NRI^+^ and NRI^–^. Confidence intervals were calculated by bootstrapping. Net reclassification improvement was determined for addition of sVCAM-1 levels in model prediction of AF and of HF in several models. This included a basic model, which included age at inclusion, sex, BMI, diabetes, hypertension, CAD, smoking, status, proteomic batch, and model A, which further included NT-proBNP levels. For AF, we conducted a secondary analysis incorporating genetic risk, in which the cohort was stratified by tertiles of AF PRS (low, intermediate, and high), with NRI assessed separately within each subgroup comparing model A with a model further adjusted for sVCAM-1.

To estimate absolute risk of AF and HF we calculated crude cumulative incidences using the Aalen–Johansen estimator (prodlim package in R), considering all-cause mortality as a competing risk. The cohort was stratified by sVCAM-1 tertiles (low, intermediate, and high).

In the sub-cohort with cMRI and subsequent sVCAM-1 measurements available, we used multivariable linear regression models to assess sVCAM-1’s association with cardiac remodelling. All models were adjusted for sex and the following covariables at the time of imaging: age, BMI, diabetes, hypertension, CAD, smoking status, and imaging centre.

Statistical analyses were conducted in R (version 4.1.0). We adjusted for multiple testing using a Bonferroni correction [*P* < 0.0071 (0.05/7 tests)] were considered statistically significant.

## Results

**Figure 1:**
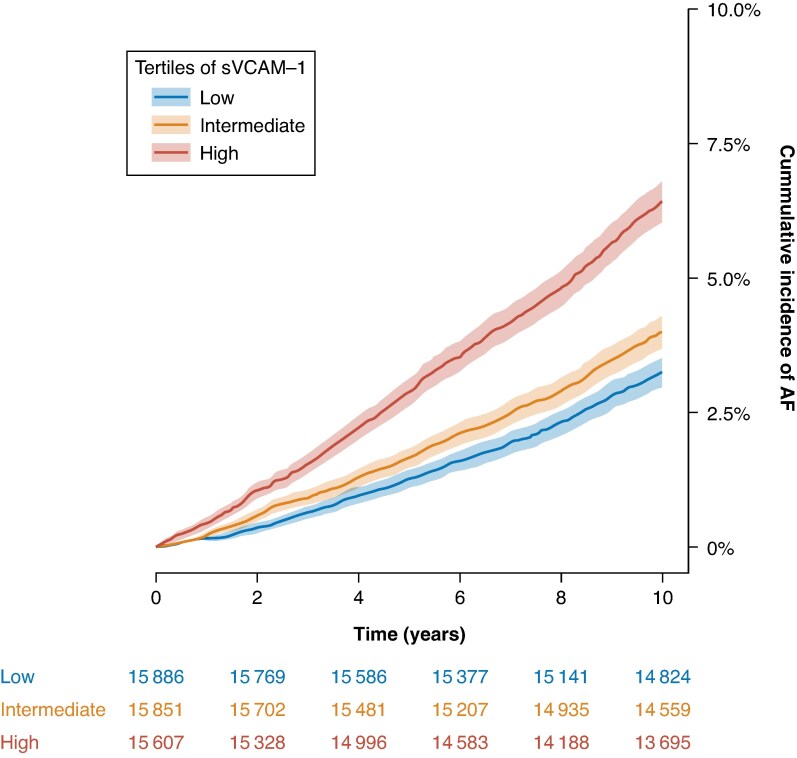
Cumulative incidence of AF according to tertiles of sVCAM-1. Crude cumulative incidence curves for AF stratified by tertiles of sVCAM-1 levels. Blue denotes the lowest tertile, yellow the intermediate tertile, and red the highest tertile. Shaded bands indicate 95% confidence interval. AF, atrial fibrillation.

**Figure 2:**
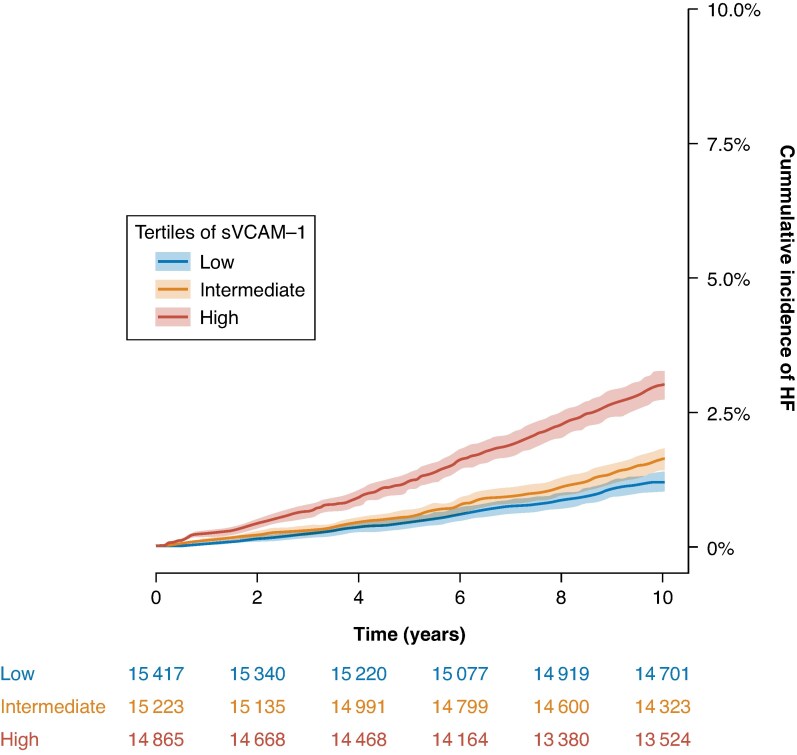
Cumulative incidence of HF according to tertiles of sVCAM-1. Crude cumulative incidence curves for HF stratified by tertiles of sVCAM-1 levels. Blue denotes the lowest tertile, yellow the intermediate tertile, and red the highest tertile. Shaded bands indicate 95% confidence interval. HF, heart failure.

**Figure 3:**
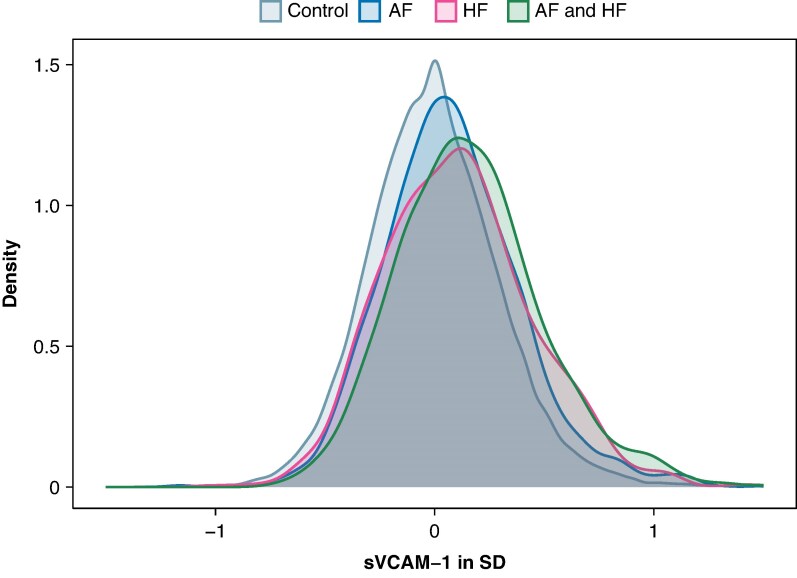
Distribution of sVCAM-1 levels (normalised to SD) in individuals with AF, HF, control and with concomitant AF and HF. The grey colour depicts the distribution of s-VCAM-1 levels in control individuals without AF or HF. The blue colour depicts the distribution of sVCAM-1 levels in individuals with incident atrial fibrillation (AF) without concomitant heart failure (HF), pink colour depicts the distribution of sVCAM-1 levels in individuals with heart failure (HF) without concomitant AF, green colour depicts the distribution of sVCAM-1 levels in individuals with both AF and HF. The x-axis represents sVCAM-1 levels normalized to standard deviations (SD). The y-axis represents the density of observations.

This study included 48 495 individuals (54.6% women) with a median age of 58.0 (first to third quartile range 50.0–63.0) years. Baseline characteristics at inclusion in the UK Biobank are summarized in *Table 1*. Cumulative incidences are visualized in *Figure 1* for AF and *Figure 2* for HF and summarized at 10 years of follow-up in *Table 2*. As illustrated in *Figure 3*, density plots demonstrated differences in the distribution of sVCAM-1 levels across individuals with AF, HF, controls, and concomitant AF and HF. A shift towards higher values of sVCAM-1 was visually apparent in individuals with AF, HF, and most pronounced in those with concomitant AF and HF.

**Table 1:** Baseline characteristics at enrolment and imaging

	Primary study cohort (*n* = 48 495)	Imaging sub-cohort (*n* = 933)
Sex		
Female, *n* (%)	26 480 (54.6)	507 (54.3)
Male, *n* (%)	22 015 (45.4)	426 (45.7)
Age, years, median (1st–3rd quartile)	58.0 (50.0–63.0)	58.3 (53.9–64.7)
BMI, kg/m^2^, mean (SD)	27.4 (4.8)	26.6 (4.4)
Comorbidities		
Diabetes, *n* (%)	2565 (5.3)	36 (3.9)
Hypertension, *n* (%)	13 185 (27.2)	209 (22.4)
Coronary artery disease, *n* (%)	2496 (5.1)	35 (3.8)
Smoking status, *n* (%)		
Never	26 569 (54.8)	598 (64.1)
Previous	16 787 (34.6)	295 (31.6)
Current	5139 (10.6)	40 (4.3)

Baseline characteristics at index date for the entire study cohort with sVCAM-1 measurements, and for the sub-cohort used for cardiac remodelling, respectively. Characteristics are at the time of inclusion for the entire cohort and for the time of imaging visit for the imaging sub-cohort. BMI, body mass index, n, number, SD, standard deviation.

**Table 2:** Ten years cumulative incidences for AF and HF across tertiles of sVCAM-1

Tertile	Cumulative incidence AF	Cumulative incidence HF
Low	3.25% (95% CI: 2.98–3.53)	1.19% (95% CI: 1.02–1.36)
Intermediate	4.00% (95% CI: 3.69–4.30)	1.62% (95% CI: 1.42–1.82)
High	6.44% (95% CI: 6.05–6.82)	3.01% (95% CI: 2.74–3.29)

Cumulative incidences of atrial fibrillation and heart failure at 10 years of follow-up, according to sVCAM-1 levels. Cohort was stratified into tertiles, denoted as low, intermediate, and high sVCAM-1 levels. AF, atrial fibrillation; CI, confidence interval; HF, heart failure.

### Association with atrial fibrillation and heart failure

**Figure 4:**
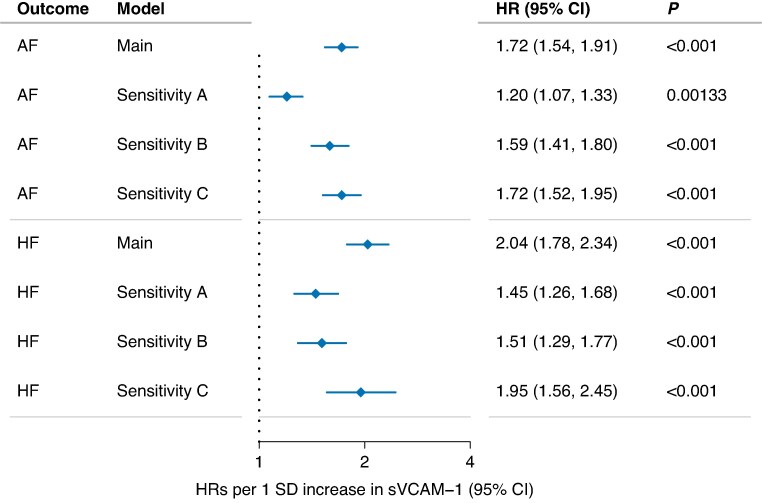
sVCAM-1 association with incident atrial fibrillation and heart failure. Hazard ratios for sVCAM-1’s association with incident atrial fibrillation and heart failure. Hazard ratios are per 1 SD increase. Main model is a multivariable Cox regression adjusted for sex, age at inclusion, body mass index, diabetes, hypertension, coronary artery disease, smoking status. and proteomic batch. Estimates for sensitivity-A model are adjusted as main model plus NT-proBNP. Estimates for sensitivity B are adjusted as main model plus CRP, leukocytes, TNF, and IL-6. Sensitivity C is a cause-specific Cox regression for incident atrial fibrillation, heart failure, and coronary artery disease adjusted as main model. CI, confidence interval; HR, hazard ratios; P, *P*-value; SD, standard deviation. HRs and CIs are presented on a log scale.

During a median follow-up of 13.7 years (first to third quartile 12.9–14.5), 3484 individuals were diagnosed with AF, while 1937 individuals were diagnosed with HF over a median follow-up period of 13.8 years (first to third quartile 13.0–14.5). Concomitant AF and HF were observed in 975 individuals; of these, AF was followed by HF in 487 individuals, HF was followed by AF in 239 individuals, and same-day diagnoses occurred in 249 individuals. One SD increase in sVCAM-1 was associated with higher rates of both incident AF [HR: 1.72, 95% confidence interval (CI): 1.54–1.91, *P* < 0.001] and incident HF (HR: 2.04, 95% CI: 1.78–2.34, *P* < 0.001). Estimates were attenuated, but remained statistically significant, when adjusting for NT-proBNP in sensitivity model A (AF; HR: 1.20, 95% CI: 1.07–1.33, *P* = 0.0013 and HF; HR: 1.45, 95% CI: 1.26–1.68, *P* < 0.001) and inflammation markers in sensitivity model B (AF; HR: 1.59, 95% CI: 1.41–1.80, *P* < 0.001 and HF; HR: 1.51, 95% CI: 1.29–1.77, *P* < 0.001). In sensitivity model C, we performed a cause-specific Cox regression with CAD as a competing event. This did not substantially alter the results for AF (HR: 1.72, 95% CI: 1.52–1.95, *P* < 0.001) or HF (HR: 1.95, 95% CI: 1.56–2.45, *P* < 0.001) (*Figure 4*).

In sub-analyses with concomitant HF and AF as time-dependent covariates, respectively, one SD increase in sVCAM-1 was associated with AF (HR 1.67; 95% CI 1.50–1.86; *P* < 0.001), and with HF (HR 1.88; 95% CI 1.65–2.15; *P* < 0.001), respectively. Incident AF was associated with higher rates of subsequent HF (HR 4.90; 95% CI 4.27–5.62; *P* < 0.001), and incident HF was associated with higher rates of subsequent AF (HR 6.11; 95% CI 5.48–6.82; *P* < 0.001).

**Figure 5:**
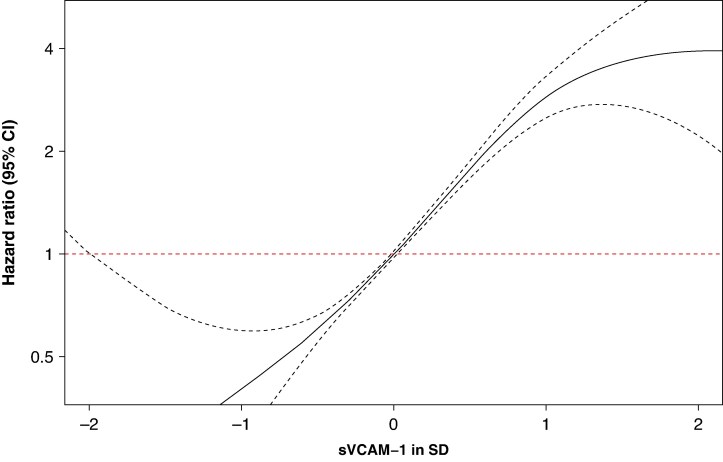
Penalized spline model of atrial fibrillation risk according to sVCAM-1 levels normalized to standard deviation (SD). Penalized spline model of atrial fibrillation risk according to normalized sVCAM-1 levels (in standard deviations, SD). P-nonlinearity: 0.018. The *x*-axis depicts sVCAM-1 levels in SD, while the y-axis shows the hazard ratio (HR) on a log scale. The solid black line represents the estimated HR, and the dashed lines indicate the 95% confidence intervals (CIs). The red dashed horizontal line corresponds to an HR of 1.

**Figure 6:**
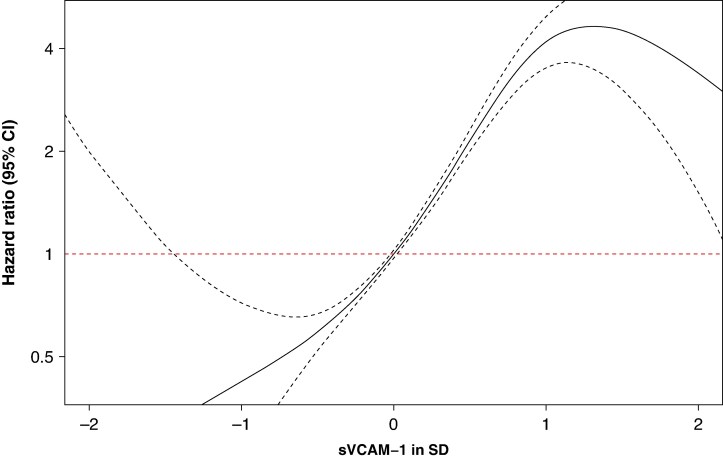
Penalized spline model of heart failure risk according to sVCAM-1 levels normalized to standard deviation (SD). Penalized spline model of heart failure risk according to normalized sVCAM-1 levels (in standard deviations, SD). P-nonlinearity: 3.4 × 10^−5^. The x-axis depicts sVCAM-1 levels in SD, while the y-axis shows the hazard ratio (HR) on a log scale. The solid black line represents the estimated HR, and the dashed lines indicate the 95% confidence intervals (CIs). The red dashed horizontal line corresponds to an HR of 1.

We examined sVCAM1 as a continuous measure by penalized splines. Visual inspection of the spline plots suggested an approximately linear relationship with a trend towards increasing HR for both outcomes as plasma levels of sVCAM1 increased (*Figures 5* and *6*). Testing, however, showed evidence of non-linearity for both outcomes (P-nonlinearity: 0.018 for AF, P-nonlinearity: 3.4 × 10^−5^ for HF).

Pearson correlation analyses showed that sVCAM-1 was modestly correlated with NT-proBNP (Pearson *r* = 0.22, *P* < 0.001) and TNF (Pearson *r* = 0.36, *P* < 0.001), and weakly correlated with leukocytes (Pearson *r* = 0.02, *P* < 0.001), CRP (Pearson *r* = 0.09, *P* < 0.001) and IL-6 (Pearson *r* = 0.16, *P* < 0.001).

### Risk of AF according to genetic predisposition and s-VCAM-1 levels

**Figure 7:**
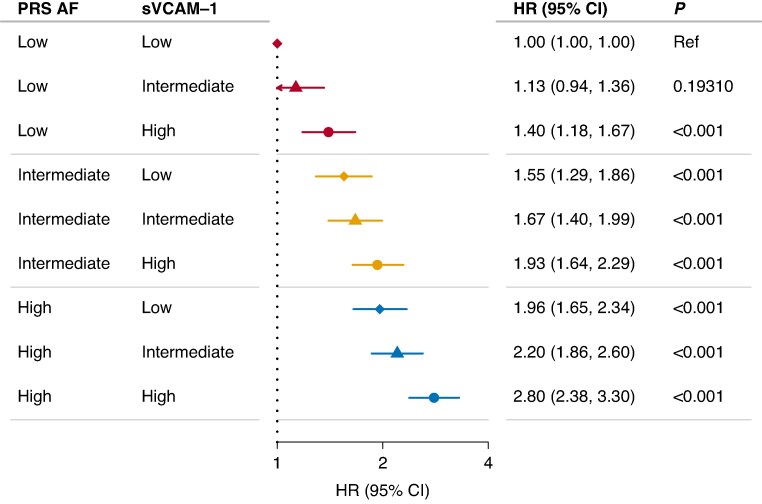
Aggregate risk of AF according to genetic predisposition and sVCAM-1 levels. Hazard ratios of incident AF according to genetic predisposition and sVCAM-1. The tertiles of sVCAM-1 are represented by symbols: diamond (low), triangle (intermediate), and circle (high). The tertiles of AF PRS are indicated by colour: red (low), yellow (intermediate), and blue (high). The model is adjusted for sex, age at inclusion, BMI, diabetes, hypertension, coronary artery disease, smoking status and proteomic batch. CI, confidence interval; HR, hazard ratio; P, *P*-value. HRs and CIs are presented on a log scale.

We assessed the aggregate risk of AF in a multivariable Cox model stratified on tertiles of PRS and sVCAM-1 (*Figure 7*). All combinations showed a statistically significant increase in HR of AF compared with the reference group (low PRS and low sVCAM-1), except group 2 (low PRS and intermediate sVCAM-1). We observed a dose–response-like relationship of AF risk with the highest rates of incident AF observed in individuals with both a PRS and sVCAM1 levels in the top tertiles [HR: 2.80, 95% confidence interval (CI): 2.38–3.30, *P* < 0.001, *Figure 7*].

### Reclassification of AF and HF according to sVCAM-1

For incident AF, the addition of sVCAM-1 resulted in an NRI of 16% (95% CI: 9.5–22.9) compared to the basic model and an NRI of 2.2% (95% CI: −4.3 to 8.4) compared to model A. For incident HF, the addition of sVCAM-1 resulted in an NRI of 21% (95% CI: 10.6–30.3) compared to the basic model and an NRI of 9.2% (95% CI: −1.2 to 18.5) compared to model A. For the NRI analysis stratified by AF and PRS, the NRI for incident AF was 0.09% (95% CI: −6.4 to 6.3) in the low group, 2.8% (95% CI: −4.0 to 9.8) in the intermediate group, and 5.0% (95% CI: −1.3 to 11.2) in the high group.

### Association with cardiac remodelling

**Figure 8:**
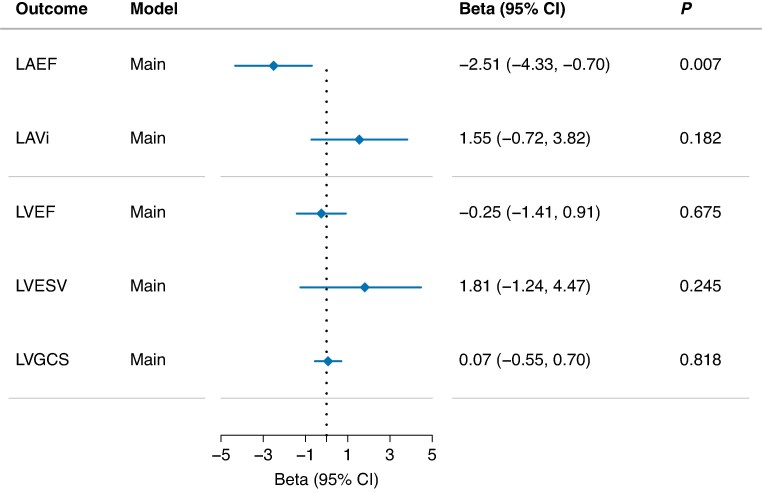
sVCAM-1 association with cardiac remodelling. Beta estimates for sVCAM-1’s levels per 1 SD increase. LA_EF_, LV_EF_, and LV_GCS_ were measured in percentage (%). LV_ESV_ were measured in millilitre (mL). LAVi was measured in mL per square metre (mL/m^2^). Estimates for the main model is adjusted for sex, age at imaging, body mass index, diabetes, hypertension, coronary artery disease, smoking status, and imaging centre. CI, confidence interval; LA_EF_, left atrial emptying fraction; LAVi, left atrial volume index; LV_EF_, left ventricular ejection fraction; LV_ESV_, left ventricular end systolic volume; LV_GCS_, left ventricular global circumferential strain; P, *P*-value. Beta estimates and CIs are presented on a linear scale.

In the sub-cohort with available cardiac imaging data from 933 individuals (54.3% women), median age of imaging was 58.3 (first to third quartile range 53.9–64.7) years. Of the two left atrial measures, higher levels of sVCAM-1 were significantly associated with LA_EF_ (β: −2.51, 95% CI: −4.33 to −0.70, *P* = 0.007) but not with LAVi (β: 1.55, 95% CI: −0.72 to 3.82, *P* = 0.182). No association was found between sVCAM-1 and measures of the left ventricle: LV_EF_ (β: −0.25, 95% CI: −1.41 to 0.91, *P* = 0.675), LV_ESV_ (β: 1.81, 95% CI: −1.24 to 4.47, *P* = 0.245), and LV_GCS_ (β: 0.07, 95% CI: −0.55 to 0.70, *P* = 0.818) (*Figure 8*).

## Discussion

This study leveraged large-scale clinical, proteomic, and imaging data from the UK Biobank to thoroughly evaluate the dynamic inflammation biomarker sVCAM-1 as a marker of AF and HF risk. In this study, we showed an association between sVCAM-1 and increased rates of AF and HF. The association remained robust across sensitivity analysis, adjusting for the well-established cardiac biomarker NT-proBNP and a selected panel of key biomarkers of systemic inflammation (CRP, leukocytes, TNF, and IL-6), and in sensitivity analysis considering CAD as a competing event. Furthermore, the association remained robust in sub-analyses accounting for concomitant HF and AF as time-dependent covariates, respectively. Further integrating genetic predisposition for AF revealed a dose–response-like relationship, with the highest rates of AF in individuals within the top tertiles of PRS and sVCAM-1 levels. We also observed that higher levels of sVCAM-1 were significantly associated with reduced LA_EF_ but not with other cardiac measures.

### Inflammation in AF and HF

The European Society of Cardiology currently recommends measuring NT-proBNP in the diagnosis of HF.^14^ Although NT-proBNP is recognized as the gold standard biomarker of AF and HF, it has some limitations.^15^ Moreover, as AF and HF are characterized by complex pathophysiological mechanisms,^1,14^ relying on single biomarkers may not adequately capture the complete disease process.

Accumulating evidence supports an important role of inflammation in the pathophysiology of AF^31–33^ and inflammation is also recognized as part of the pathogenesis of HF.^34^ CRP is a well-known yet nonspecific inflammatory marker induced by IL-6.^35^ Higher levels of CRP are associated with increased risk of incident AF and AF reoccurrence^36,37^ and have also been demonstrated to be elevated in HF patients.^15^ The inflammation biomarkers IL-6 and TNF have also been associated with AF.^38,39^ sVCAM-1 is a more specific biomarker of inflammation and may aid in improved risk stratification of AF and HF, and it potentially offers more insight into the pathophysiologic mechanism of the diseases.

The role of sVCAM-1 is to mediate leukocyte adhesion and transmigration through the endothelia of the blood vessels,^19,20^ and its expression is upregulated in response to proinflammatory cytokines such as TNF-alfa. It is recognized as an active contributor to inflammation in the development of atherosclerosis.^21^ In our study, we showed that elevated levels of sVCAM-1 at baseline were associated with increased rates of AF. This is consistent with the accumulating evidence of inflammation as part of the pathophysiology of AF. A study by Oyama *et al*.^40^ demonstrated that serial measurements of hsTnT, NT-proBNP, and GDF-15 in patients with AF provided increased prognostic value beyond the baseline levels. In our study, only measurement of sVCAM-1 at baseline was available; however, it would be of interest in future studies to assess whether serial assessment of sVCAM-1 would provide additional information.

In the present study, we associated sVCAM-1 as a biomarker of both AF and HF development. This is consistent with recent findings from another cohort, which reported an association between sVCAM-1 and HF with reduced ejection fraction.^41^ sVCAM-1 might be superior to CRP as a biomarker of AF and HF development, as elevations in sVCAM-1 are specific to endothelial inflammation, whereas CRP is a more nonspecific inflammatory marker. Our Pearson correlation test showed that sVCAM-1 was only weakly correlated with CRP, thus implying sVCAM-1 elevations as an expression of a distinct inflammatory pathway.

A study by González-Ferrero *et al.*^42^ measured galectin-3, fatty acid-binding protein-4 (FABP4), and soluble receptor for advanced glycation end products (sRAGE) and found no differences in biomarker levels between individuals with AF alone and those with concomitant heart failure. In our study, we found that sVCAM-1 levels were higher in individuals with concomitant AF and HF than in those with AF or HF alone. This may hint that sVCAM-1 captures a shared disease pathway for AF and HF.

Since sVCAM-1 is an inflammatory biomarker and has been linked to atherosclerotic disease^21^ and as CAD is a known risk factor associated with AF,^1^ we performed a sensitivity analysis further adjusting for a selected panel of other more general inflammatory biomarkers (CRP, leukocytes, TNF, and IL-6) and conducted a sensitivity analysis with CAD as a competing event, to ensure that our results were not entirely driven by inflammation associated with CAD, or through shared pathways with the panel of inflammatory biomarkers. The association of increasing levels of sVCAM-1 and AF and HF development remained robust across these sensitivity analyses, confirming that the observed associations were not solely driven by underlying systemic inflammation or CAD. Future studies are warranted to determine the potential of sVCAM-1 in a clinical setting.

### Aggregate risk of genetic predisposition and sVCAM-1

Currently, there is a lack of consensus and clear guidelines on who should undergo PRS testing and when it is relevant in a clinical setting. Notably, the current ESC guidelines on cardiovascular disease prevention do not recommend routine PRS of atherosclerotic cardiovascular disease,^43^ reflecting knowledge gaps in genetic risk stratification. However, the integration of PRS in a clinical setting is thought to have the potential to improve risk prediction,^44^ and the integration of AF PRS with existing clinical prediction models has demonstrated significant improvements in risk prediction of AF.^45^ An advantage of PRS is that it is stable throughout an individual’s lifespan and only needs to be assessed once.^44^ It is thought that the potential benefit of accessing AF PRS particularly lies in risk prediction in the high-risk groups and among young individuals without comorbidities or other AF risk factors.^45^

A PRS does not translate to a direct pathophysiological mechanism but rather captures a lifelong increased susceptibility to AF. Plasma measures of sVCAM-1 represent a dynamic biomarker of inflammation known to rise with age.^46^ We integrated the lifelong genetic risk through AF PRS with sVCAM-1 to assess the aggregate risk of genetic predisposition and sVCAM-1. Higher levels of sVCAM-1 were associated with increased rates of AF across all strata of genetic risk. The observed dose–response-like relationship indicates that integrating genetic risk could aid in identifying high-risk individuals who may benefit the most from screening and early interventions. A recent study by Ahn *et al.*^47^ found that an HF PRS did significantly improve the risk prediction of incident HF in individuals with AF, and this was particularly pronounced among those younger than 60 years. This aligns with the known increased risk of incident cardiovascular events and mortality following AF^4^ and could indicate that our result with increased risk in the individuals with high PRS and high sVCAM-1 levels, could be of importance, especially in younger individuals. Whereas Ahn *et al.* focused on genetic predisposition captured by PRS, our combined risk model extends this approach by additionally accounting for dynamic inflammatory risk through sVCAM-1. Furthermore, we demonstrated that sVCAM-1 was associated with AF risk across strata of genetic predisposition, supporting its potential broader utility as a biomarker beyond genetic high-risk individuals.

### Cardiac remodelling

Atrial myopathy, defined by the European Society of Cardiology as “*Any complex of structural, architectural, contractile or electrophysiological changes affecting the atria with the potential to produce clinically-relevant manifestations*”^48^ is increasingly recognized as a substrate of AF, HF, and stroke.^49–51^ Inflammatory cell infiltration is thought to contribute to the development of an arrhythmogenic substrate for AF.^48^ We integrated data from cMRI to assess associations between sVCAM-1 and cardiac remodelling in individuals with no prior diagnosis of AF or HF at the time of inclusion in the UK Biobank. We observed that higher levels of sVCAM-1 were associated with lower LA_EF_, which could be indicative of a developing structural change and potentially even atrial myopathy in patients with increased levels. A study by Mathew *et al*.^52^ found that sVCAM-1 was associated with left atrial peak longitudinal strain, also implying that sVCAM-1 somehow mediates inflammation within the atrium. They also found an association with LV_GCS_; however, we found no association with any ventricular measures. Interestingly, we found no association between sVCAM-1 and LV_EF_, although we found an association with HF. Atrial myopathy has increasingly been recognized as a possible substrate of HF with preserved ejection fraction.^53–55^ Our results indicate that sVCAM-1 may reflect processes related to early atrial remodelling (i.e. atrial cardiomyopathy).^53^ However, the observed association with LA_EF_ should be interpreted with caution, as the subgroup for this analysis consisted of 933 individuals, given that both sVCAM-1 measurements and cMRI data had to be available. The absence of associations with other atrial measures may reflect limited statistical power. Further investigation in larger cohorts is warranted.

### Clinical implications

This is the first large-scale cohort study examining sVCAM-1 as a biomarker of AF and HF development. Our findings suggest that sVCAM-1 has potential as a biomarker in AF and HF screening. We also observed a dose–response-like relationship in the aggregate risk analysis, integrating AF PRS. sVCAM-1 was more strongly associated with AF development in high-risk individuals and might be valuable as a biomarker in high-risk individuals. Thus, in future studies, it would be interesting to investigate the application of aggregate risk, particularly in younger individuals aged 40–60 years without known comorbidities or other established AF risk factors. This group, which is not captured by conventional clinical risk models, has been shown to have an increased risk of incident cardiovascular events following AF diagnosis.^4^ It would be relevant to explore whether those with a high combined risk of PRS and sVCAM-1 might benefit from intensified heart rhythm monitoring to prevent adverse outcomes. However, consensus on the implementation of PRS in clinical practice is still lacking.^44^

Considering the accumulating evidence of the role of inflammation in the pathophysiology of AF and HF, our study provides insights into sVCAM-1 as an inflammatory marker of AF and HF risk. NRI analysis showed a significant improvement in classification in the basic model when including sVCAM-1 levels. However, in model A, which was further adjusted for NT-proBNP levels, the reclassification improvement was attenuated and not statistically significant. This finding may reflect the strong prognostic value of NT-proBNP in AF, which leaves limited room for improvement by additional markers. Nevertheless, in the multivariate cox regression sVCAM-1 remained associated with both AF and HF despite adjusting for NT-proBNP levels. While NT-proBNP reflects myocardial stretch, sVCAM-1 levels may provide additional pathophysiological insight into the inflammatory component of AF development. We also found a negative association with LA_EF_, which may reflect processes linked to early atrial myopathy.

Measurements of sVCAM-1 could potentially help stratify patients according to disease aetiology, guiding the clinician in individualized screening and therapeutic approach. Recent studies have also highlighted sVCAM-1 as a potential marker of aging, and levels of sVCAM-1 have been shown to rise with age in both mice and humans.^46^ Furthermore, decreased levels of sVCAM-1 have been associated with increased lifespan in humans.^56^ An animal model study found that aged mice injected with antibodies against VCAM-1 resulted in improved cognitive function.^46^ In future studies, it would be of interest to explore whether the association with sVCAM-1 differs across AF phenotypes, including paroxysmal, persistent, and permanent AF, as well as across HF phenotypes, such as HF with reduced and preserved ejection fraction. Our findings provide a foundation for future research into the clinical applicability of sVCAM-1 as a biomarker of inflammation in AF and HF.

### Limitations

The UK Biobank population is primarily of European ancestry, and the results may therefore not apply to other populations. The setup of the UK Biobank may induce healthy volunteer bias.^55^ The detection of incident AF is dependent on the methods applied, and therefore the prevalence in this study may underestimate the true occurrence of AF.^56^ Another limitation of this study is that sVCAM-1 was only assessed at baseline (and at imaging time for the cMRI subgroup analysis). Consequently, we lack temporal information about sVCAM-1 and cannot account for individual fluctuations. The analysis of cardiac remodelling was limited to investigation in a sub-cohort (*n* = 933) since measurements of both sVCAM-1 and cMRI had to be available. UK Biobank does not provide phenotypic information on HF, thereby preventing differentiation between HF with reduced and preserved ejection fraction. It is important to note that the protein measurements of sVCAM-1 in the UK Biobank were standardized, and therefore, it is not possible to provide a clinical threshold value. Future studies should aim to establish relevant cut-off values for the implementation of sVCAM-1 in a clinical setting, while also assessing whether normalized rather than absolute protein concentrations may be sufficient to define clinically relevant thresholds. Given the observational study design of the UK Biobank, residual confounding remains a potential source of bias, and causality cannot be assumed.

## Conclusion

In more than 48 000 individuals with plasma proteomic measures, sVCAM-1 was associated with increased rates of AF and HF, aligning with previous smaller cohort studies. The associations remained consistent after adjustment for other markers of inflammation, and in sensitivity analyses considering CAD as a competing event. Higher levels of sVCAM-1 were associated with a reduced LA_EF_, hinting at a potential role in adverse atrial remodelling, while integration with a genetic risk score identified a risk gradient for AF across both genetic risk and sVCAM-1 levels. Further investigation is necessary to determine clinically meaningful thresholds and potential applications in screening, risk stratification, and treatment.

## Supplementary Material

euaf246_Supplementary_Data

## Data Availability

All data from this study is available through the UK Biobank. Access to the UK Biobank is available for bona fide researchers through https://www.ukbiobank.ac.uk/enable-your-research/apply-for-access.
