# Supplementary material for: Vascular cell adhesion molecule 1: a marker for atrial fibrillation and heart failure risk
Source: Europace. 2025 Oct 25;27(10):euaf246. doi: 10.1093/europace/euaf246 (PMC12569766; doi:10.1093/europace/euaf246)
Supplement: euaf246_Supplementary_Data [file euaf246_supplementary_data.docx]

**Supplemental material**

**Baadsgaard *et al.*** Vascular cell adhesion molecule 1: a marker for atrial fibrillation and heart failure risk

Index

[Supplemental Table 1. Datafields and phenotype definitions 2](#_Toc212102457)

[Supplemental Table 2. Interaction analyses 3](#_Toc212102458)

[Supplemental Figure 1. Flowchart – participant selection 4](#_Toc212102459)

## Supplemental Table 1. Datafields and phenotype definitions

| **Phenotype/variable** | **UK Biobank data field(s)** | **Corresponding ICD10 code(s)** |
| --- | --- | --- |
| Sex | 31, 22001 | - |
| Age at inclusion | 21003 | - |
| Death | 40000 | - |
| Ethnic background | 21000 | - |
| Body-mass index | 21001 | - |
| Atrial fibrillation | 131350 | I48 |
| Diabetes | 130706, 130708, 130714 | E10, E11, E14 |
| Heart failure | 131354 | I50 |
| Hypertension | 131286 | I10 |
| Coronary artery disease | 131296, 131298, 131300, 131304, 131306 | I20, I21, I22, I24, I25 |
| Smoking status | 20116 | - |
| Plasma leukocytes | 30000 | - |
| Plasma creatinine | 30700 | - |
| Plasma proteins | Category 1839 | - |
| Left atrial emptying fraction | 24113 | - |
| Left atrial maximum volume | 24110 |  |
| Left ventricular ejection fraction | 24103 | - |
| \|  \| Left ventricular circumferential strain global \| \| --- \| --- \| | 24157 | - |
| Left ventricular end systolic volume | 31062 | - |

## Supplemental Table 2. Interaction analyses

| **Outcome** | **Interaction** | **HR** | **CI lower** | **CI higher** | **P** |
| --- | --- | --- | --- | --- | --- |
| AF | sVCAM-1*age | 0.983412 | 0.9667 | 1.0004 | 0.055846 |
| AF | sVCAM-1*sex | 0.845016 | 0.6850 | 1.0424 | 0.115861 |
| AF | sVCAM-1*AF-PRS | 1.320650 | 1.2778 | 1.3649 | 2×10^-16^ |
| HF | sVCAM-1*age | 1.006930 | 0.9846 | 1.0298 | 0.5464 |
| HF | sVCAM-1*sex | 0.811846 | 0.6215 | 1.0605 | 0.1262 |

The model was adjusted as main model. AF, atrial fibrillation. CI, confidence interval. HF, heart failure. HR, hazard ratio. P, p-value. PRS, polygenic risk score.

## Supplemental Figure 1. Flowchart – participant selection
